# CD103^+^ tumor-infiltrating lymphocytes predict favorable prognosis in patients with esophageal squamous cell carcinoma

**DOI:** 10.7150/jca.30354

**Published:** 2019-08-28

**Authors:** Yifan Chu, Jing Liao, Jinqing Li, Yongchun Wang, Xingjuan Yu, Junfeng Wang, Xiue Xu, Liyan Xu, Limin Zheng, Jing Xu, Lian Li

**Affiliations:** 1 State Key Laboratory of Oncology in South China, Collaborative Innovation Center for Cancer Medicine, Sun Yat-sen University Cancer Center, Guangzhou 510060, P. R. China; 2 MOE Key Laboratory of Gene Function and Regulation, School of Life Sciences, Sun Yat-sen University, Guangzhou 510275, P. R. China; 3 The Key Laboratory of Molecular Biology for High Cancer Incidence Coastal Chaoshan Area, Shantou University Medical College, Shantou 515041, China

**Keywords:** esophageal squamous cell carcinoma, prognosis, CD103, tumor-infiltrating lymphocytes

## Abstract

As an indispensable factor in preventing the recirculation of tissue lymphocytes to the lymphatic and blood systems, the integrin CD103 has enabled the characterization of lymphocyte populations in non-lymphoid tissues and organs. However, the expression, distribution, and clinical significance of CD103^+^ tumor-infiltrating lymphocytes (TILs) in esophageal squamous cell carcinoma (ESCC) remain unclear. In the present study, we included tumor and adjacent non-tumor tissue specimens from 198 patients with ESCC who had undergone surgical resection. Immunohistochemistry and immunofluorescence were used to detect CD103^+^ TIL distribution, as well as the co-expression of CD103 and T cell markers and functional molecules. Kaplan-Meier analysis and the Cox proportional hazards model were used to estimate the prognostic value of CD103^+^ TILs. The results showed that CD103^+^ TILs were predominantly located in adjacent non-tumor tissues compared with tumor tissues (*P* < 0.0001). Immunofluorescence double staining revealed that CD8^+^ T cells, but not CD4^+^ T cells, comprised the majority of CD103-expressing cells. Most of these CD103-expressing cells co-expressed CTLA-4 and granzyme B rather than the exhaustion marker PD-1. High density of intratumoral CD103^+^ TIL is associated with longer overall survival (OS) and disease-free survival (DFS) in both the internal (OS, *P* = 0.0004 and DFS, *P* = 0.0002) and external (OS, *P* = 0.038 and DFS, *P* = 0.12) cohorts. Multivariate Cox analysis showed the density of CD103^+^ TILs was an independent positive prognostic factor for OS (hazards ratio [HR] = 0.406; *P* = 0.0003 in the internal cohort; HR = 0.328, *P* = 0.01, in the external cohort) and DFS (HR = 0.385; *P* = 0.0002 in the internal cohort; HR = 0.270, *P* = 0.003, in the external cohort). Our findings indicate that CD103^+^ TILs might play an important role in the tumor microenvironment, and intratumoral CD103^+^ TILs could serve as a promising prognostic marker in ESCC.

## Introduction

Esophageal cancer is one of the leading causes of cancer-related death worldwide 1. The majority of cases in China is esophageal squamous cell carcinoma (ESCC) 2. Although there has been progressive improvement in surgical resection and neoadjuvant chemoradiotherapy, the 5-year survival rate of patients with ESCC remains unsatisfactory 3. Therefore, there is an urgent need to develop novel clinical strategies for treating patients with ESCC.

Recently, immunotherapies targeting immune checkpoints have been proven to be promising treatments across different cancers 4-6, including esophageal carcinoma 7. Tumor responses to these therapies are mediated mainly by anti-tumor T cell immunity previously blocked by CTLA-4 (cytotoxic T lymphocyte-associated protein 4) or PD-1 (programmed cell death 1). Accordingly, clinical trials have observed increased T cell frequency in many patients who had received immunotherapy 8. Despite the evaluation of T cell counts, only a minority of patients have objective tumor responses, indicating that the number of tumor-infiltrating lymphocytes (TILs), which comprise complex subpopulations with diverse activities, may be inadequate to reflect the efficacy of anti-tumor immunity. Therefore, identifying the T cell subpopulations involved in anti-tumor immunity and that influence disease progression should be helpful in clinical decisions on patient treatment.

CD103 is the αE integrin subunit of the heterodimeric αEβ7 complex whose ligand is the epithelial cell surface molecule E-cadherin 9. CD103 expression has been found on different immune cells, such as dendritic cells (DC) 10, CD8^+^ cytotoxic T cells 11, and CD4^+^ T cells 12. The adhesive interaction of CD103 with E-cadherin can mediate T cell recruitment and the localization of antigen-specific T cells in the lung and skin 13, 14. Recent studies have found that increased CD103^+^ tissue-resident CD8^+^ T cells are associated with improved survival in melanoma 8, lung cancer 15, and cervical cancer 16, suggesting that CD103^+^ T cells are a potential predictive biomarker. Furthermore, it has been reported that TILs like CD8^+^ T cells correlated with improved survival in ESCC. However, some studies showed conflicting results 17, 18. Therefore, there is an urgent need to identify an accurate marker that can determine the prognosis of TILs for enabling the development of novel clinical strategies for treating patients with ESCC.

In the present study, we analyzed the distribution, composition, and prognostic significance of CD103^+^ TILs in patients with ESCC. The majority of CD103-expressing cells were CD8^+^ T cells, and they could express CTLA-4 and granzyme B (GB). Moreover, high CD103^+^ cell numbers predicted favorable prognosis in patients with ESCC.

## Materials and Methods

### Patients and tissue specimens

Archived, formalin-fixed, paraffin-embedded tumor samples were obtained from patients with pathologically confirmed ESCC at the Sun Yat-sen University Cancer Center (SYSUCC) in 2002-2009 and at the Shantou Central Hospital (STCH) in 2010-2011. One hundred and seven patients from SYSUCC and ninety-one patients from STCH who had not received anti-tumor therapy preoperatively were enrolled randomly in the study. The patients' clinicopathological parameters were obtained from medical records and pathology reports. All tumor stages were determined according to American Joint Cancer Committee/Union International Center Cancer (AJCC/UICC) classification guidelines. ESCC specimen grading and histopathology subtyping was based on World Health Organization criteria. Patient consent was obtained prior to the use of the clinical materials for research purposes. The study was approved by the Institutional Review Boards of the participating hospitals, and it was implemented in strict accordance with the ethical guidelines of the Declaration of Helsinki.

### Follow-up

Follow-up was performed with regular surveillance for recurrence using imageological examination at 2-4-month intervals. Disease-free survival (DFS) was defined by the time from diagnosis to death or relapse, whichever occurred first, or to the last follow-up date. Overall survival (OS) was defined as the interval between the time of surgery and either death or the last observation. Table 1 summarizes the patients' clinicopathological parameters.

### Immunohistochemistry (IHC) and immunofluorescence staining

The formalin-fixed, paraffin-embedded samples were cut into 5-μm sections and subjected to IHC and immunofluorescence staining as described previously 19-21. Briefly, tissue sections were incubated with primary antibodies against CD103 (rabbit anti-human, ab129202, Abcam, Cambridge, UK), CD8 (rabbit anti-human, MA5-14548, Thermo Fisher Scientific, Waltham, MA, USA), CD4 (mouse anti-human, ZM-0418, Zhongshan Bio-Tech, Guangdong, China), PD-1 (mouse anti-human, ZM-0381, Zhongshan Bio-Tech), CTLA-4 (mouse anti-human, 14-1529-80, eBioscience, San Diego, CA, USA), and CD11c (rabbit anti-human, ab52632, Abcam). Immunostaining was performed using horseradish peroxidase-conjugated anti-rabbit and anti-mouse antibodies from DAKO EnVision systems (Dako Cytomation, Glostrup, Denmark) and was developed with peroxidase and 3, 3′-diaminobenzidine tetrahydrochloride. All sections were counterstained with Mayer's hematoxylin and mounted in non-aqueous mounting medium.

For double immunofluorescence staining of CD3, CD11c, CD4, CD8, CTLA-4 or PD-1, and CD103, we used species-paired fluorescently labeled secondary antibodies [donkey anti-rat immunoglobulin G (IgG) (H+L) secondary antibody, Alexa Fluor 488, A-21206; donkey anti-mouse IgG (H+L) secondary antibody, Alexa Fluor 555, A-31570, Invitrogen, Waltham, MA, USA] and tyramide reagent (Alexa Fluor 488 Tyramide Reagent, B40953; Alexa Fluor 555 Tyramide Reagent, B40955, Invitrogen). Nuclei were counterstained using 4′, 6-diamidino-2-phenylindole (DAPI).

### Image analysis

To evaluate CD103^+^ cell density, we used the Vectra-InForm image analysis system (Perkin-Elmer/Applied Biosystems, Foster City, CA, USA) as described previously 22, 23. For IHC, the five most representative high-power fields were captured at ×200 magnification (0.284 mm^2^ per field) for each tumor region in all specimens. CD103^+^ cells in each field were counted and analyzed manually by two independent observers blinded to clinical outcome. Positively stained cells with morphological features characteristic of lymphocytes were counted based on localization in the intratumoral (IT) and adjacent non-tumor (ANT) regions. Data are reported as the mean (± SEM) number of cells per field.

Immunofluorescence images were captured using a confocal microscope (Olympus, Essex, UK) and analyzed using a FV10-ASW Viewer (Olympus). Two independent observers blinded to the outcome counted and analyzed single- or double-positive cells in each of five representative fields at ×400 magnification (0.07 mm^2^ per field). Data are reported as the mean (± SEM) number of cells per field.

### Statistical analyses

All statistical analyses were performed using SPSS version 20.0 (SPSS Inc., Chicago, IL, USA). The significance of differences between groups was determined by the Wilcoxon signed rank test. Survival curves were calculated by the Kaplan-Meier method and analyzed by the log rank test. The Cox proportional hazards model was used to identify prognostic factors through univariate and multivariate analyses. The statistical significance of differences between groups was determined using the two-tailed Student *t*-test, where *P* < 0.05 was considered statistically significant.

## Results

### Study population

Table 1 summarizes the clinicopathological features of the patients. The average follow-up time was 38.0 months (range, 2.2-123.7 months) in SYSUCC cohort and 45.7 months (range, 3.0-84.8 months) in STCH cohort. During the follow-up, 121 patients (65.2%) died. Altogether, we enrolled 150 men and 48 women; their average survival time was 41.2 months and 41.7 months, respectively. Of all patients, 57.1% were > 55 years old. Based on the seventh edition of the AJCC staging manual, 22 cases (11.1%) were histologically graded as poorly differentiated, 162 cases (81.8%) as moderately or well-differentiated, and 14 cases (7.1%) as deficient. Among these patients, 19 (9.6%) had AJCC pathologic stage I disease and 179 (90.4%) had stage II-IV disease.

### Distribution of CD103^+^ cells

**Figure 1:**
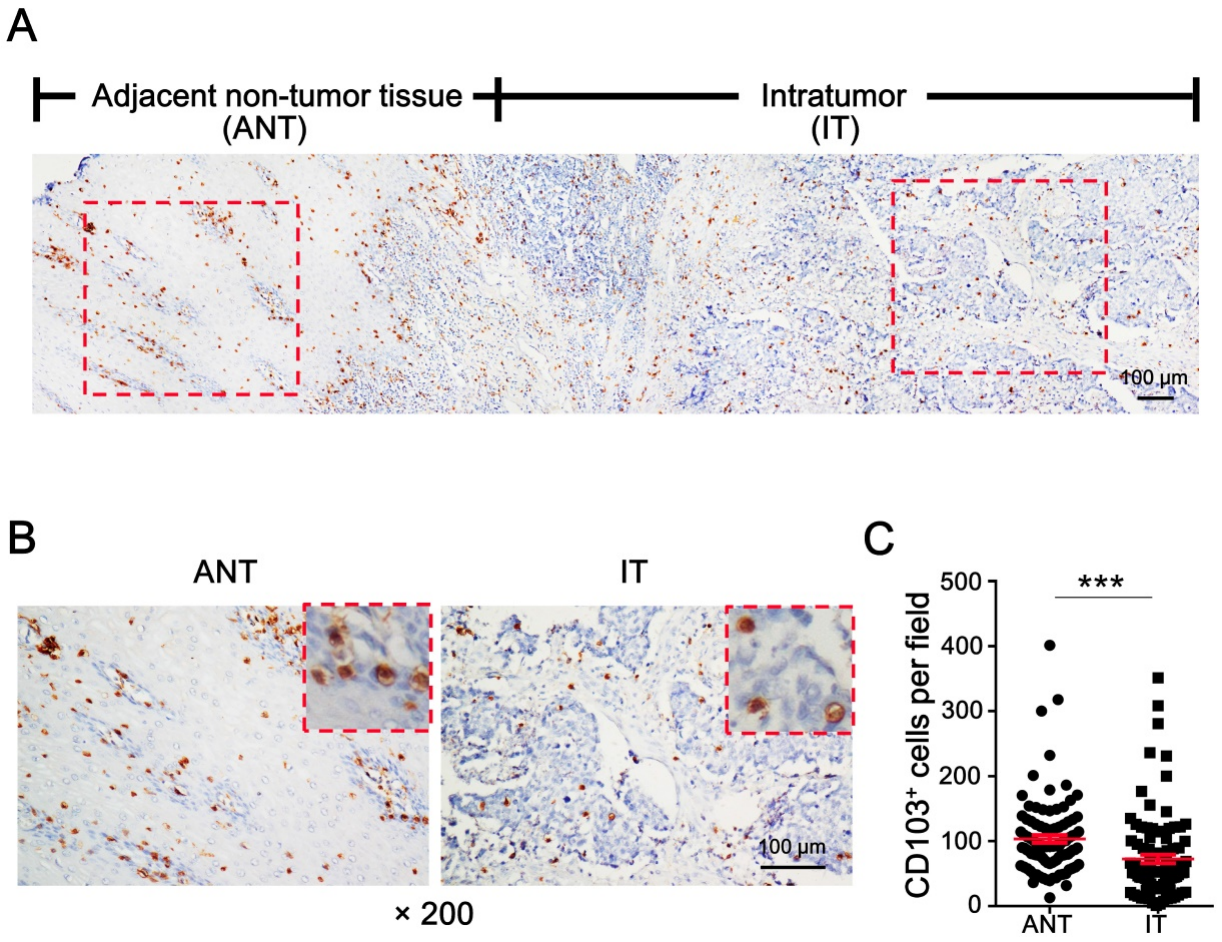
** CD103^+^ cells are decreased in ESCC tumor tissue.** (A) IHC staining shows CD103 in adjacent non-tumor tissue (ANT) and intratumor (IT) regions in ESCC tissue specimen. Scale bar = 100 μm. (B) Enlargements of the outlined areas in (A) showing outlined areas at higher magnification. Scale bar = 100 μm. (C) Quantification of CD103^+^ cell densities in the ANT and IT regions (*n* = 95). Results are the mean ± SEM (bars); ***P < 0.001.

To evaluate the distribution of CD103^+^ cells in ESCC, tumor specimens containing the advancing edges of intratumoral (IT) and adjacent non-tumor (ANT) regions were used for IHC staining (Fig. 1A). Diffusely distributed CD103^+^ cells were detected in both the IT and ANT regions (Fig. 1A, 1B). CD103^+^ cell density was significantly higher in the ANT tissue (mean ± SEM: 103 ± 6 cells/field) than in the IT tissue (mean ± SEM: 73 ± 7 cells/field; *P* < 0.0001; Fig. 1C). These data suggest that CD103^+^ cell numbers might decrease during the transition from epithelial to tumor tissue.

### Cellular source of CD103^+^ cells

**Figure 2:**
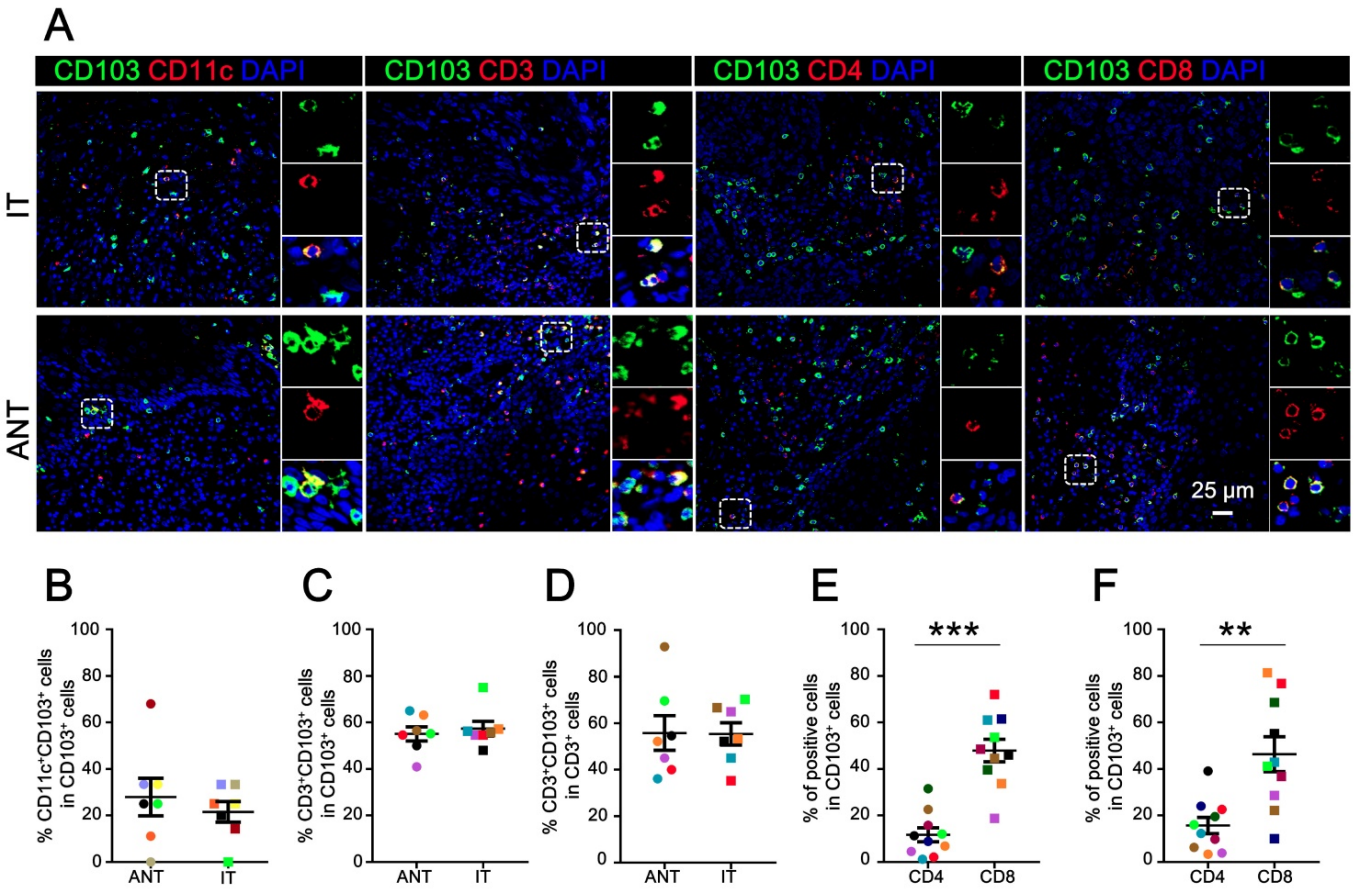
** Cellular source of CD103^+^ cells in ESCC tissues.** (A) Double immunofluorescence staining shows CD103 (green), CD3 (red), CD11c (red), CD4 (red), and CD8 (red) expression and co-localization of double-positive cells (yellow) in ESCC tissue. (B) The percentages of CD11c^+^CD103^+^ cells identified as CD11c and CD103 double-positive cells and calculated in total numbers of CD103^+^ cells in the ANT and IT regions. Scale bar = 25 μm. (C, D) The percentages of CD3^+^CD103^+^ cells identified as double-positive cells and calculated in total numbers of CD103^+^ or CD3^+^ cells in the ANT and IT regions (*n* = 7). (E, F) The percentages of CD4^+^CD103^+^ and CD8^+^CD103^+^ cells identified as double-positive cells and compared to the total numbers of CD103^+^ cells in the ANT (E) and IT regions (F), respectively (*n* = 10). Results are the means ± SEM (bars); **P < 0.01; ***P < 0.001.

CD103^+^ cells are a heterogeneous population of cells 24. Multicolor immunofluorescence staining was performed to characterize the cellular source of CD103^+^ cells in ESCC. As expected, CD103^+^ cells co-expressed CD11c in both the ANT (mean ± SEM: 27.97 ± 8.08%) and IT regions (mean ± SEM: 21.56 ± 4.42%) (Fig. 2A, 2B). Notably, we detected a high frequency of CD3^+^CD103^+^ cells in the total CD103^+^ cells from both the ANT (mean ± SEM: 55.04 ± 3.06%) and IT regions (mean ± SEM: 57.29 ± 3.16%) (Fig. 2A, 2C). Moreover, these CD3^+^CD103^+^ cells comprised about half of the CD3^+^ cells in the ANT (mean ± SEM: 55.75 ± 7.45%) and IT regions (mean ± SEM: 55.36 ± 4.79%) (Fig. 2D).

To identify the cellular source of CD103^+^ TILs, co-localization immunofluorescence was performed to calculate the proportion of CD4^+^CD103^+^ and CD8^+^CD103^+^ cells in the total CD103^+^ cells (Fig. 2A). In the IT tissue, the CD8^+^CD103^+^/CD103^+^ cell ratio (mean ± SEM: 46.35 ± 7.50%) was significantly higher than the CD4^+^CD103^+^/CD103^+^ cell ratio (mean ± SEM: 15.69 ± 3.50%; *P* < 0.01; Fig. 2F). The ANT region followed a similar trend (mean ± SEM: CD8^+^CD103^+^/CD103^+^ cell ratio, 47.93 ± 4.83%; CD4^+^CD103^+^/CD103^+^ cell ratio, 11.68 ± 3.00%; *P* < 0.001; Fig. 2E). Collectively, our findings indicate that the primary CD103-expressing cells in ESCC tissues are CD8^+^ T cells.

### Phenotype of CD103^+^ TILs

**Figure 3:**
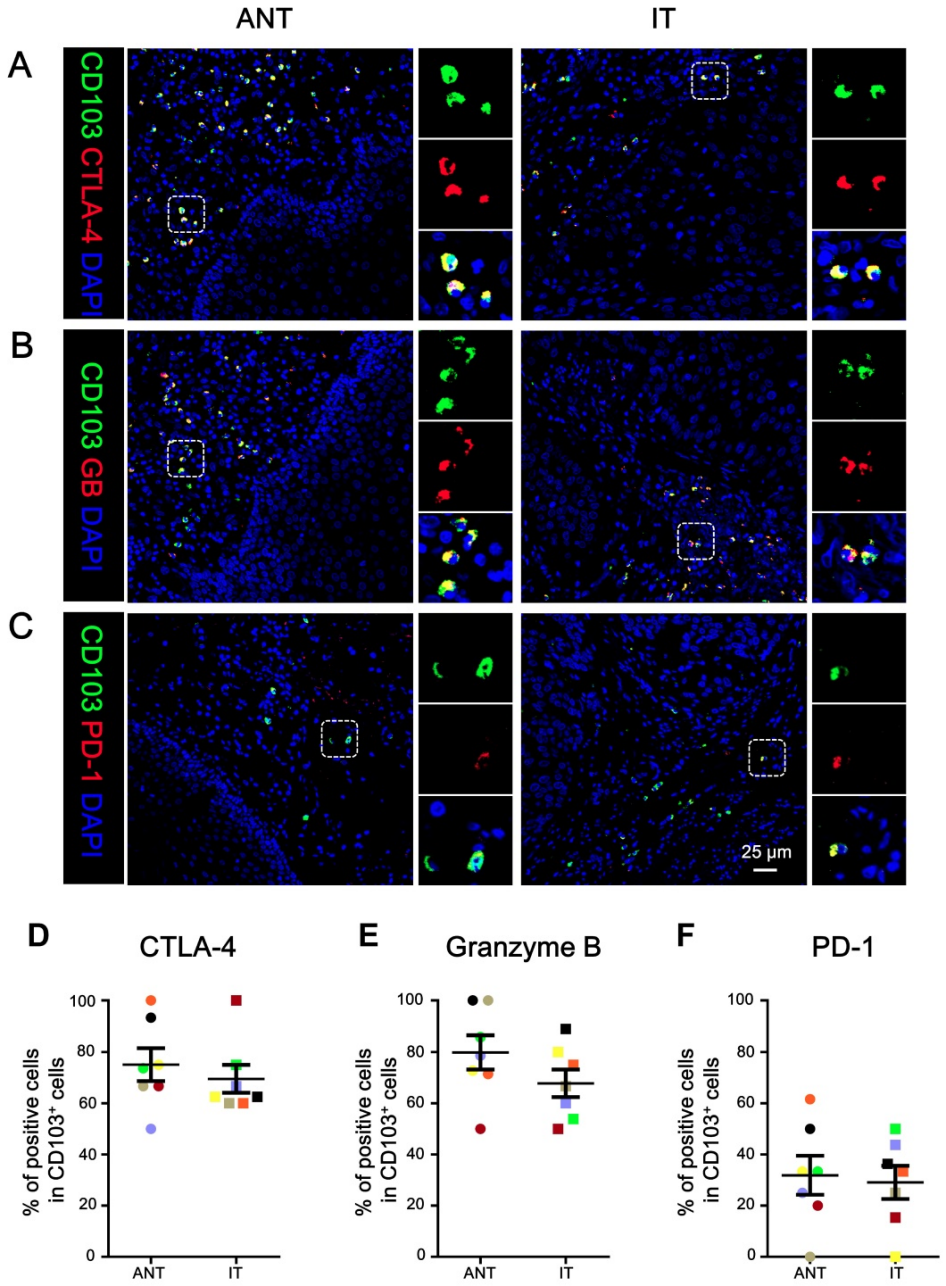
** CD103^+^ cells co-express CTLA-4, granzyme B (GB), and PD-1 in ESCC.** (A-C) Double immunofluorescence staining shows CD103, CTLA-4 (A), granzyme B (GB) (B), and PD-1 (C) expression and co-localization of double-positive cells in ESCC tissue. DAPI (blue) was used as a counterstain. Scale bar = 25 μm. The percentages were identified as double-positive cells compared to the total numbers of CD103^+^ cells in the ANT and T regions, respectively (*n* = 7). Results are the means ± SEM (bars).

We determined the *in-situ* phenotype of the CD103^+^ TILs. Immunofluorescence staining showed that the majority of CD103^+^ cells were positive for CTLA-4 (mean ± SEM: ANT, 75.05 ± 6.41%; IT, 69.52 ± 5.45%; Fig. 3A, 3D) and granzyme B (mean ± SEM: ANT, 79.78 ± 6.66%; IT, 67.77 ± 5.39%; Fig. 3B, 3E). However, the CD103^+^ TILs expressed relatively lower levels of PD-1 (mean ± SEM: ANT, 31.89 ± 7.59%; IT, 29.12 ± 6.50%; Fig. 3C, 3F). Taken together, the intratumoral CD103^+^ cells had a CTLA-4^hi^GB^hi^PD-1^low^ phenotype, suggesting that CD103^+^ TILs in ESCC exhibit an activated phenotype.

### Prognostic value of CD103^+^ cells

**Figure 4:**
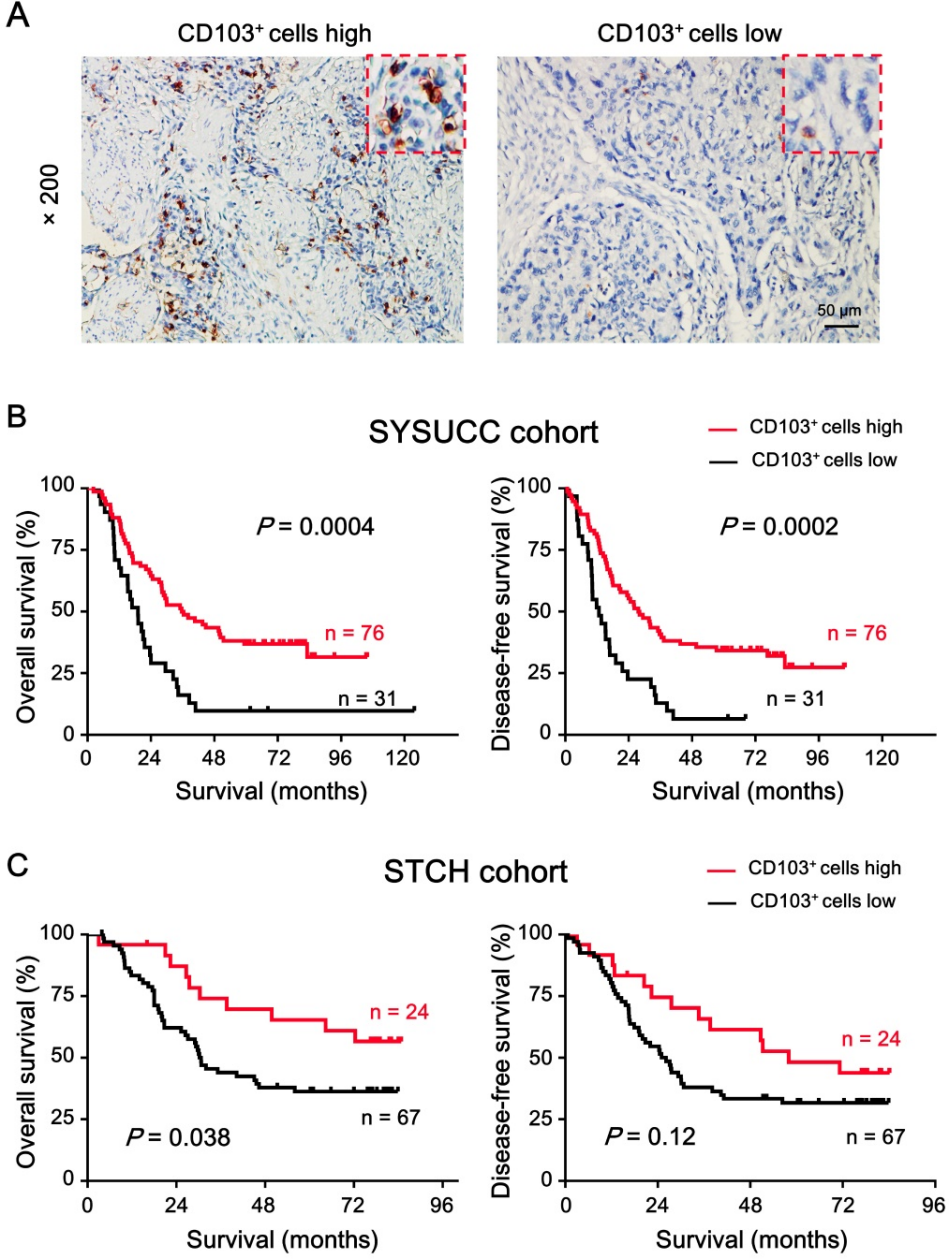
** High intratumoral CD103^+^ cell density is a predictor of favorable prognosis in patients with ESCC.** (A) Representative IHC images demonstrate high (left) and low (right) number of CD103^+^ cells. Brown areas indicate positively stained cells. Scale bar = 50 μm. (B) Kaplan-Meier survival curves compare OS and DFS rates in patients (*n* = 107) with low or high intratumoral CD103^+^ cell density in SYSUCC cohort. (C) Kaplan-Meier survival curves compare OS and DFS rates in patients (*n* = 91) with low or high intratumoral CD103^+^ cell density in STCH cohort. *P* < 0.05 was considered statistically significant (log-rank test).

To investigate the relationship between CD103^+^ cell density in the ESCC tumor region and patient survival, we divided the patients into two groups based on the receiver operating characteristic. The CD103^+^ cell density cut-off value was 37.58 cells/field; approximately 71% of patients (76/107) had higher CD103^+^ cell density. Kaplan-Meier survival curves plotted to investigate the association with survival showed that patients with high CD103^+^ cell density had better OS (median: 38 months, *P* = 0.0004; Fig. 4A) and DFS (median: 32 months, *P* = 0.0002; Fig. 4B) than patients with low CD103^+^ cell density. The 5-year OS and DFS rates for patients with high CD103^+^ cell density were 34.21% and 34.21%, respectively, while that for patients with low CD103^+^ cell density were 9.67% and 12.9%, respectively. We also analyzed the correlation between CD103^+^ cell density and patient clinicopathological features. Table 2 shows that intratumoral CD103^+^ cell number was not significantly correlated with patient gender, age, T classification, lymph node metastasis, clinical staging, differentiation, or metastasis in both cohorts.

To further validate the prognostic role of CD103^+^ cell density, an independent external cohort of patients with pathologically confirmed ESCC from STCH was enrolled. The correlation of clinicopathological parameters between the two cohorts is showed in Table 1. The cut-off value derived from the internal cohort was applied to the external cohort. The results showed that patients with high CD103^+^ cell density had better OS (mean: 58.9 months, *P* = 0.038) and DFS (mean: 51.1 months, *P* = 0.12) as compared to patients with low CD103^+^ cell density (mean OS: 41.0 months; mean DFS: 36.0 months) in the external cohort (Fig. 4C).

Variables that were associated with survival by univariate analysis were adopted as covariates in the multivariate Cox model. Table 3 showed that high CD103^+^ cell density was associated with better OS (hazard ratio [HR] = 0.406, 95% confidence interval [CI] = 0.247-0.667, *P* = 0.0003) and DFS (HR = 0.385, 95% CI = 0.233-0.637, *P* = 0.0002) in the internal set, as well as in the external cohort (HR = 0.328, 95% CI = 0.141-0.763, *P* = 0.01; DFS: HR = 0.270, 95% CI = 0.113-0.644, *P* = 0.003). Therefore, CD103^+^ cell density could serve as an independent prognostic factor of OS and DFS in patients with ESCC.

## Discussion

CD103^+^ cells are an important component of inflammatory infiltrating lymphocytes in tissue, where they exhibit diverse subpopulations and a distinct phenotype 24-26. In the present study, we delineated the distribution and prognostic value of CD103^+^ cells in ESCC tissue. Intratumoral tissues had decreased CD103^+^ cell density compared with non-tumor tissues. CD8^+^ T cells accounted for most CD103^+^ cells in the ESCC tissue and exhibited a CTLA-4^hi^GB^hi^PD-1^low^ activated phenotype. Furthermore, we demonstrated that intratumoral CD103^+^ cell density was associated with favorable prognosis and could be served as an independent risk factor in patients with ESCC.

The heterogeneity of T cells is responsible for their diverse functions in tumor progression 27-29. Previous studies have found that clinical significance of T cell subsets remains controversial 30-34. Therefore, there is an urgent need to identify accurate prognostic factors to enable the development of novel clinical strategies for treating patients with ESCC. CD103 is a convenient cell surface marker for tissue-resident memory T cells 26, 35. We observed that most CD103^+^ cells were CD3^+^ T cells in ESCC tissues and the intratumoral tissues had decreased CD103^+^ cell density compared to the non-tumor tissues. Moreover, high CD103^+^ TILs density was associated with better OS and DFS. Multivariate analyses revealed that CD103^+^ TILs number was an independent and significant prognostic factor in ESCC. In accordance with our results previous studies showed that CD103^+^ TIL density has been associated with improved prognosis in patients with non-small cell lung cancer 15, endometrial adenocarcinoma 36, ovarian cancer 37, cervical cancer 16, and bladder cancer 11. Recent study has found that five features (MYC, ANO1, SLC52A3, Age and N-stage, MASAN) provided a precise prediction of ESCC survival outcome 38. The combination of CD103 (ITGAE) with MASAN may has the potential to be further studied as the optimal predictor for ESCC prognosis. Moreover, the applicability of these cells across heterogeneous populations of ESCC patients should be further validated.

TILs subsets span the innate-adaptive continuum and include innate lymphoid cells, unconventional T cells (e.g., natural killer T cells, γδ T cells), and tissue-resident memory T cells, which have diverse phenotypes and functions in homeostasis, infection, and tumors 39. Many TILs express CD103, which binds E-cadherin, mediating cellular localization in tumor tissue 9. The CD103^+^CD39^+^ CD8 TILs identified tumor-reactive CD8 T cells in many human solid tumor 40. Upon infection, in contrast to CD103^-^CD8^+^ T cells, most CD103^+^CD8^+^ cells can quickly upregulate cytotoxic mediators when exposed to their specific antigen, which might provide a rapid and efficient response to influenza infection without causing cytotoxic damage to the epithelial barrier 41. Moreover, the CD11b^-^CD103^+^PD-L1^high^ DCs in mesenteric lymph nodes (MLNs) could take up intestinal luminal antigens, and highly induce Foxp3^+^ regulatory T cells through TGF-β activation 42. Accordingly, we observed that most CD103^+^ TILs were CD8^+^ T cells, which accounted for about 46% of such cells in the ESCC tissues; CD11c^+^ DC made up fewer of the numbers, suggesting that the CD8^+^CD103^+^ subset in CD103^+^ TILs play a primary role in the anti-tumor immune response. Therefore, the underlying mechanisms that regulate CD103^+^ TILs subset migration, location, and development, including their diverse functions, which local environmental signals might influence, warrant further investigation.

In a normal physiological environment, immune checkpoints are crucial for maintaining self-tolerance 43. CTLA-4 is expressed mainly on T cells, where it primarily regulates the amplitude of the early stages of T cell activation 44. On the other hand, when exposed to chronic antigens, such as viral infection and tumor, T cells are educated to a state of exhaustion or dysfunction, with increased PD-1 expression, which limits their lytic activity 45. In the present study, CD103^+^ TILs displayed a CTLA-4^hi^GB^hi^PD-1^low^ activated phenotype in the tumor. In cervical cancer,* ITGAE* gene expression has been strongly correlated with cytotoxic T cell markers (e.g., CD8/granzyme B), and in a preclinical mouse model, human papillomavirus (HPV) E6/E7 targeted therapeutic vaccination combined with radiotherapy increased the number of intratumoral CD103^+^CD8^+^ T cells, indicating that CD103 is a promising response biomarker of HPV E6/E7 targeted immunotherapy 16. We found that most CD103^+^ TILs co-located with CTLA-4^+^ cells, but confocal microscopic analysis showed that fewer co-expressed PD-1 *in situ*. Moreover, the majority of CD103^+^ TILs secreted granzyme B, which could kill the target cells. Taken together, our results suggest that CD103^+^ TILs have anti-tumor potential and are deserved in-depth mechanism research on their anti-tumor activity, which might have functional roles in cancer immunotherapy.

## Conclusions

In ESCC, CD103^+^ cells are mainly activated, and not exhausted T cells that exhibit a CTLA-4^hi^GB^hi^PD-1^low^ phenotype. The density of CD103^+^ TILs predicts favorable prognostic value during tumor progression. These results can be used as a promising prognostic biomarker for patients with ESCC.

## Figures and Tables

**Table 1:** Clinicopathological characteristics of the patients

Characteristics	Total	SYSUCC	STCH	*P* value^*^
	Cases (%)	Cases (%)	Cases (%)	
***Gender***				
Male	150 (75.8)	80 (74.8)	70 (76.9)	0.724
Female	48 (24.2)	27 (25.2)	21 (23.1)	
***Age (year)***				
> 55	113 (57.1)	53 (49.5)	60 (65.9)	0.02
≤ 55	85 (42.9)	54 (50.5)	31 (34.1)	
***T Classification***				
T3 - T4	138 (69.7)	63 (58.9)	75 (82.4)	***P* < 0.001**
T1 - T2	60 (30.3)	44 (41.1)	16 (17.6)	
***N Classification***				
Yes	78 (39.4)	43 (40.2)	35 (38.5)	0.804
No	120 (60.6)	64 (59.8)	56 (61.5)	
***Recurrence***				
Yes	77 (38.9)	19 (17.8)	58 (63.7)	***P* < 0.001**
No	121 (61.1)	88 (82.2)	33 (36.3)	
***TNM Staging***				
Ⅱ-Ⅳ	179 (90.4)	100 (93.5)	79 (86.8)	0.114
Ⅰ	19 (9.6)	7 (6.5)	12 (13.2)	
***Differentiation***^†^				
Poor	22 (12.0)	13 (12.1)	9 (11.0)	0.736
Well + Moderate	162 (88.0)	89 (83.2)	73 (89.0)	

Abbreviations: TNM, tumor-node-metastasis. SYSUCC, Sun Yat-sen University Cancer Center. STCH, Shantou Central Hospital. ^†^ Data was missing in these variables for some patients.^*^P values represent the correlation on clinicopathological features between SYSUCC cohort and STCH cohort.

**Table 2:** Correlation between the density of CD103^+^ cells and clinicopathological parameters

Characteristic		CD103^+^ Cells					
		SYSUCC			STCH		
		low	high	*P* value	low	high	*P* value
		cases (%)	cases (%)		cases (%)	cases (%)	
Gender	Male	23 (74.2)	57 (75.0)	0.931	51 (76.1)	19 (79.2)	0.761
	Female	8 (25.8)	19 (25.0)		16 (23.9)	5 (20.8)	
Age (years)	> 55	13 (41.9)	40 (52.6)	0.315	43 (64.2)	17 (70.8)	0.555
	≤ 55	18 (58.1)	36 (47.4)		24 (35.8)	7 (29.2)	
T classification	T3 - T4	21 (67.7)	42 (55.3)	0.234	55 (82.1)	20 (83.3)	0.582
	T1 - T2	10 (32.3)	34 (44.7)		12 (17.9)	4 (16.7)	
N classification	yes	11 (35.5)	32 (42.1)	0.526	27 (40.3)	7 (29.2)	0.333
	no	20 (64.5)	44 (57.9)		40 (59.7)	17 (70.8)	
Recurrence	yes	4 (12.9)	15 (19.7)	0.578	45 (67.2)	13 (54.2)	0.256
	no	27 (87.1)	61 (80.3)		22 (32.8)	11 (45.8)	
TNM staging	Ⅱ-Ⅳ	30 (96.8)	70 (92.1)	0.671	58 (86.6)	21 (87.5)	0.608
	Ⅰ	1 (3.2)	6 (7.9)		9 (13.4)	3 (12.5)	
Differentiation	poor	6 (19.4)	7 (9.9)	0.186	7 (11.7)	6 (27.3)	0.086
	well + moderate	25 (80.6)	64 (90.1)		53 (88.3)	16 (72.7)	

*P* values were analyzed by χ^2^ test or Fisher's exact test, as appropriate.

**Table 3:** Univariate and multivariate analyses of variables associated with overall survival and disease-free survival in the internal and external cohort

variables	OS								DFS						
	univariate				multivariate				univariate				multivariate		
	HR	95% CI	*P* value		HR	95% CI	*P* value		HR	95% CI	*P* value		HR	95% CI	*P* value
***SYSUCC cohort***															
Gender (male/female)	0.781	0.456-1.338	0.368						0.866	0.518-1.449	0.585				
Age (>55/≤55)	1.053	0.673-1.648	0.821						0.953	0.616-1.475	0.829				
T classification (T3-T4/T1-T2)	1.571	0.979-2.521	0.061						1.587	0.998-2.522	0.051				
N classification (yes/no)	2.050	1.307-3.215	**0.002**		2.265	1.416-3.623	**0.001**		1.879	1.209-2.919	**0.005**		1.657	1.030-2.664	**0.037**
Recurrence (yes/no)	1.646	0.976-2.773	0.061						2.143	1.265-3.630	**0.005**		2.146	1.190-3.869	**0.011**
TNM staging (Ⅱ-Ⅳ/Ⅰ)	4.041	0.991-16.478	0.051						4.246	1.042-17.306	**0.044**				**N.S.**
Differentiation (poor/well + moderate)	2.138	1.141-4.007	**0.018**		1.952	1.023-3.726	**0.043**		2.038	1.094-3.798	**0.025**		2.318	1.206-4.455	**0.012**
CD103^+^cells (low/high)	0.432	0.269-0.694	**0.001**		0.406	0.247-0.667	**0.0003**		0.421	0.264-0.670	**0.0003**		0.385	0.233-0.637	**0.0002**
≥ 37.6 cells/field*															
< 37.6 cells/field															
***STCH cohort***															
Gender (male/female)	1.335	0.669-2.661	0.412						1.764	0.891-3.490	0.103				
Age (>55/≤55)	1.382	0.766-2.494	0.283						1.325	0.759-2.312	0.323				
T classification (T3-T4/T1-T2)	2.103	0.897-4.930	0.087						2.609	1.118-6.088	**0.026**				**N.S.**
N classification (yes/no)	2.488	1.436-4.309	**0.001**				**N.S.**		2.745	1.624-4.642	**0.0002**				**N.S.**
Recurrence (yes/no)	63.692	8.743-463.987	**0.0004**		60.56	8.032-456.629	**0.00006**		153.010	17.363-1348.412	**0.0001**				**N.S.**
TNM staging (Ⅱ-Ⅳ/Ⅰ)	6.019	1.462-24.780	**0.013**				**N.S.**		4.714	1.471-15.106	**0.009**				**N.S.**
Differentiation (poor/well + moderate)	0.763	0.323-1.803	0.538						1.089	0.511-2.323	0.825				
CD103^+^cells (low/high)	0.492	0.246-0.982	**0.044**		0.328	0.141-0.763	**0.01**		0.615	0.331-1.142	0.123		0.270	0.113-0.644	**0.003**
≥ 37.6 cells/field*															
< 37.6 cells/field															

*Each number represents mean value/field of all cases analyzed. Univariate analysis, Cox proportional hazards regression model. Multivariate analysis, Cox proportional hazards regression model. Variables were adopted by univariate analysis. Abbreviations: OS, overall survival; DFS, disease-free survival; HR, hazard ratio; CI, confidence interval. N.S., not significant.
